# RNA extraction from ten year old formalin-fixed paraffin-embedded breast cancer samples: a comparison of column purification and magnetic bead-based technologies

**DOI:** 10.1186/1471-2199-8-118

**Published:** 2007-12-21

**Authors:** Alfredo Ribeiro-Silva, Haiyu Zhang, Stefanie S Jeffrey

**Affiliations:** 1 Department of Surgery, Stanford University School of Medicine, MSLS Bldg Room P214; 1201 Welch Road, Stanford, CA 94305-5494, USA; 2 Department of Pathology, Ribeirão Preto Medical School, University of São Paulo, Avenida Bandeirantes 3900, Campus Universitário Monte Alegre, 14049-900; Ribeirão Preto, SP, Brazil

## Abstract

**Background:**

The development of protocols for RNA extraction from paraffin-embedded samples facilitates gene expression studies on archival samples with known clinical outcome. Older samples are particularly valuable because they are associated with longer clinical follow up. RNA extracted from formalin-fixed paraffin-embedded (FFPE) tissue is problematic due to chemical modifications and continued degradation over time. We compared quantity and quality of RNA extracted by four different protocols from 14 ten year old and 14 recently archived (three to ten months old) FFPE breast cancer tissues. Using three spin column purification-based protocols and one magnetic bead-based protocol, total RNA was extracted in triplicate, generating 336 RNA extraction experiments. RNA fragment size was assayed by reverse transcription-polymerase chain reaction (RT-PCR) for the housekeeping gene glucose-6-phosphate dehydrogenase (G6PD), testing primer sets designed to target RNA fragment sizes of 67 bp, 151 bp, and 242 bp.

**Results:**

Biologically useful RNA (minimum RNA integrity number, RIN, 1.4) was extracted in at least one of three attempts of each protocol in 86–100% of older and 100% of recently archived ("months old") samples. Short RNA fragments up to 151 bp were assayable by RT-PCR for G6PD in all ten year old and months old tissues tested, but none of the ten year old and only 43% of months old samples showed amplification if the targeted fragment was 242 bp.

**Conclusion:**

All protocols extracted RNA from ten year old FFPE samples with a minimum RIN of 1.4. Gene expression of G6PD could be measured in all samples, old and recent, using RT-PCR primers designed for RNA fragments up to 151 bp. RNA quality from ten year old FFPE samples was similar to that extracted from months old samples, but quantity and success rate were generally higher for the months old group. We preferred the magnetic bead-based protocol because of its speed and higher quantity of extracted RNA, although it produced similar quality RNA to other protocols. If a chosen protocol fails to extract biologically useful RNA from a given sample in a first attempt, another attempt and then another protocol should be tried before excluding the case from molecular analysis.

## Background

For decades, human tissue samples obtained from surgical procedures have been routinely fixed in formalin and embedded in paraffin for long-term storage. Consequently, most institutions have large paraffin-block archives that allow long-term follow up for all types of neoplasms, including rare tumors. These archives represent a valuable source for molecular studies. A major limitation for the use of such samples to study gene expression is three-fold: RNA can degrade prior to formalin fixation; formalin fixation produces significant chemical modification of RNA; and RNA continues to fragment and degrade over time, even after dehydration and paraffin-embedding.

Immediate freezing of fresh tissue samples preserves good quality RNA for gene expression studies; however, this procedure is not routinely performed in most hospitals and there are few institutions worldwide that have large frozen-tissue banks associated with long term clinical followup. Moreover, tumor samples accrued into a frozen tissue bank are inherently biased in their collection: such tumors must be sufficiently large and palpable in order for tissue to be excised and frozen for the bank [1].

Formalin-fixed paraffin-embedded (FFPE) samples are universally available and, with Institutional Review Board/Ethics Committee approval, cancer samples can be linked to clinical and/or follow up information, often available through an institution's tumor registry. Extracting RNA from such samples has been a promising but problematic challenge. In addition to RNA degradation concerns, formalin fixation creates cross-linking between nucleic acids and proteins and adds mono-methylol to amino groups for all four RNA bases (N-CH_2_OH); methylene bridging then occurs between neighboring bases (N-CH_2_-N), formed by condensation of amino bases and *N*-methylols [2].

Rupp and Locker described the first successful RNA extraction from FFPE samples in 1988 [3]. Since then, many protocols have been described [2,4-11]. There are currently multiple protocols commercially available. In most of these protocols the RNA is extracted by spin column purification according to similar basic principles: deparaffinization, followed by cell disruption with heated proteinase K, which is capable of efficiently degrading proteins that were covalently cross-linked with each other and RNA, thereby allowing more efficient RNA extraction than achieved by use of chaotropic agents such as guanidinium chloride [7]. Proteinase K incubation at high temperature (60 to 70°C) also removes part of the methylol additions induced by formalin fixation [7]. After proteinase K incubation, RNA is isolated by alcohol precipitation and though use of salt guanidine thiocyanate in a spin column purification step [2,12].

Magnetic beads can also be utilized to isolate RNA. In these protocols, nucleic acids are bonded to paramagnetic beads and isolated by a magnetic field. RNA is isolated using an optimized buffer solution and later a DNAse step. Magnetic micro-beads can be prepared in a number of ways, but usually magnetically susceptible particles (e.g. iron oxide) are coated with synthetic or biological polymers with high affinity for nucleic acid. A magnet is applied to the side of the tube containing the magnetically-labeled sample mixture and the magnetically bound nucleic acid aggregates near the wall of the tube, allowing the unlabeled materials to be pipetted off and discarded. In addition to quickly separating magnetically-bound nucleic acid, this method does not create the shear forces generated by spin centrifugation that may lead to nucleic acid degradation [13].

Breast cancer tissue RNA archived for one year is less degraded and has larger average molecular weight than RNA archived for six and 17 years, suggesting that fragmentation of FFPE tissue continues even after specimens are dehydrated and embedded in paraffin [9]. Many molecular techniques depend upon the quantity and quality of extracted RNA in samples; translational studies that link molecular findings to clinical outcome require long term follow up. Here we compare RNA extraction protocols on a set of 14 ten year old FFPE breast cancer samples, testing spin column purification and magnetic bead-based technologies. Using the same protocols, we compare our results with a second set of 14 recently archived breast cancer samples fixed and embedded in paraffin three to ten months prior to this study, that we designate "months old" samples. We also test RNA fragment size in both sample groups using RT-PCR for the housekeeping gene G6PD.

## Results

Table 1 exhibits 168 RNA extraction experiments performed on 14 ten year old archived FFPE breast cancer samples, showing RNA quantity (in pg/μl) and quality (by RNA integrity number, RIN) for RNA that was extracted in triplicate using four different extraction protocols. Similarly, Table 2 shows 168 RNA extraction experiments on 14 FFPE breast cancer samples that are months old, archived three to ten months prior to analysis, again with RNA extracted in triplicate using each of the four protocols. Tables 3 and 4 show the minimum and maximum values, means, and medians for each of the protocols concerning the quantity and the quality of the extracted RNA, in the ten year old and months old set of samples, respectively. Tables 5 and 6 show the same parameters, but only for samples that had biologically useful RNA extracted, defined as RNA with a minimum RIN of 1.4.

**Table 1:** Comparison of the four RNA extraction protocols in ten year old samples, by quantity of extracted RNA (in pg/μl) and RNA quality (by RNA integrity number, RIN). NM = not measurable.

Sample	Parameter	**Protocol 1**			**Protocol 2**			**Protocol 3**			**Protocol 4**		
		Test 1	Test 2	Test 3	Test 1	Test 2	Test 3	Test 1	Test 2	Test 3	Test 1	Test 2	Test 3

1	RNA	91,464	73,861	29,225	32,507	110,621	47,418	21,078	53,731	77,500	231,085	301,416	258,839
	RIN	NM	2.3	2.0	3.5	3.2	NM	2.2	NM	2.1	NM	2	NM
2	RNA	27,124	4,836	17,711	165,588	63,934	159,656	38,415	43,935	17,635	144,770	110,373	187,249
	RIN	1.7	1.1	2.0	2.1	NM	2.1	2.2	2.4	1.9	2.0	2.3	2.2
3	RNA	1,236	1,278	6,012	11,050	5,570	7,671	1,416	1,314	18,106	30,239	40,763	105,048
	RIN	2.3	1.0	2.2	2.3	NM	2.3	2.4	2.5	2.6	1.8	2.2	5.5
4	RNA	5,103	31,145	18,353	71,598	25,791	18,397	34,381	20,229	8,557	122,541	90,564	86,324
	RIN	NM	2.3	NM	2.2	2.9	NM	2.3	2.2	2.1	NM	NM	NM
5	RNA	5,146	2,893	7,262	43,901	5,140	8,624	29,711	14,415	11,391	68,768	72,550	76,048
	RIN	1.7	1.1	2.4	NM	2.3	2.3	NM	2.4	2.3	2.2	2.2	6.1
6	RNA	11,416	4,282	975	99,045	27,804	30,150	18,686	14,128	31,289	23,427	84,711	98,565
	RIN	NM	1.0	1.0	2.2	2.7	NM	2.4	2.4	2.1	NM	2.2	NM
7	RNA	160,254	6,878	7,584	98,598	56,896	39,390	16,501	48,935	6,070	62,394	196,847	150,394
	RIN	NM	1.0	1.1	2.2	2.5	2.2	1.2	1.0	1.1	2.6	2.2	2.2
8	RNA	65,752	2,088	9,915	66,706	28,998	18,004	46,519	12,300	31,952	12,900	87,934	85,429
	RIN	1.2	1.8	2.1	3.2	NM	NM	2.4	2.4	2.2	NM	2.2	1.9
9	RNA	15,123	15,579	20,517	32,962	21,034	265,694	137,594	65,451	46,768	101,909	132,994	95,334
	RIN	1.5	1.1	2.2	5.2	NM	NM	2.2	2.1	2.1	NM	2.3	2.1
10	RNA	31,042	36,583	12,392	68,465	26,950	25,458	35,551	74,122	11,543	35,885	76,333	42,892
	RIN	2.2	2.3	2.1	NM	2.3	2.3	3.6	4.4	2.2	NM	2.3	1.9
11	RNA	19,588	14,991	11,954	30,333	26,505	18,184	34,319	39,265	12,859	39,093	56,516	17,502
	RIN	2.2	1.4	2.1	NM	2.3	2.4	2.5	NM	1.9	2.2	2.2	2.1
12	RNA	5,883	3,042	10,918	29,701	106,627	35,362	67,125	28,579	12,037	188,026	314,693	91,516
	RIN	2	1	NM	1.5	2.1	NM	2.3	2.4	2.3	2.1	2.1	2.1
13	RNA	30,578	36,224	3,943	45,229	74,659	5,371	21,065	11,367	6,659	111,666	190,653	33,443
	RIN	2.2	2.3	1.8	NM	2.1	NM	2.2	2.3	2.1	2.1	2	NM
14	RNA	3,099	4,057	4,915	22,218	1,650	12,581	9,518	4,916	17,807	9,864	7,790	39,253
	RIN	2.3	2.5	1.9	2.4	2.3	NM	2.2	NM	2.3	2.3	NM	2.1

**Table 2:** Comparison of the four RNA extraction protocols in recently archived months old samples, by quantity of extracted RNA (in pg/μl) and RNA quality (RNA integrity number, RIN). NM = not measurable.

Sample	Parameter	**Protocol 1**			**Protocol 2**			**Protocol 3**			**Protocol 4**		
		Test 1	Test 2	Test 3	Test 1	Test 2	Test 3	Test 1	Test 2	Test 3	Test 1	Test 2	Test 3

15	RNA	26,717	75,411	4,886	13	56,455	105,945	21,028	4,606	115,194	184,832	44,758	176,904
	RIN	2.5	2.3	2.3	NM	2.3	1.7	2.3	2.3	2	2	2.4	NM
16	RNA	33,327	935	2,392	16,843	108,984	42,670	39,120	4,989	38,314	283	195,149	3,590
	RIN	NM	1.5	2.0	1.8	2.0	2.0	2.3	1.1	2.4	NM	2.1	2.6
17	RNA	12,321	15,143	24,286	16,752	94,246	16,075	4,211	73,366	59,072	25,535	102,053	48,459
	RIN	2.4	2.3	2.1	1.9	2.0	2.1	2.4	1	2.2	NM	2.0	1.9
18	RNA	151,098	2,987	54,397	45,924	201,780	34,506	57,940	9,608	92,135	34	1,430	709
	RIN	NM	2.5	1.1	2.3	1.9	2.6	2.2	2.3	2.6	1.4	2.4	1.5
19	RNA	574	5,060	8,632	44,190	6,633	9,835	7,852	15,146	12,487	5,893	177,677	38,735
	RIN	2.6	2.4	2.3	1.6	NM	2.4	2.5	2.4	2.4	2.3	2.2	2.3
20	RNA	32,152	25,444	43,271	5,011	1,943	129,140	12,681	48,499	41,880	9,862	680,783	51,245
	RIN	2.1	2.2	1.9	NM	2.1	1.9	2.1	2.1	2.3	NM	2.0	2.0
21	RNA	129	33,579	53,338	5,586	15,692	112,909	5,494	15,487	24,668	88,118	2,209,739	19,185
	RIN	NM	2.5	2.3	NM	2.5	2.3	2.4	2.5	2.4	2.3	2.1	2.5
22	RNA	10,368	28,093	8,781	20,750	47,818	337,413	10,810	21,020	13,080	86,567	1,989,467	45,987
	RIN	2.3	2.3	1.5	2.0	2.7	NM	2.2	2.4	2.4	2.2	NM	2.0
23	RNA	506	14,419	9,737	10,198	48,692	448,764	2,406	11,445	14,051	96,318	183	3,569
	RIN	NM	2.3	1.6	2.0	2.0	NM	1.4	2.3	2.3	2.0	NM	2.3
24	RNA	79,734	42,491	70,454	94,699	98,831	122,026	4,048	104,887	18,737	23,212	437,911	35,536
	RIN	NM	2.5	2.2	1.9	NM	2	2.2	2.4	2.4	2.8	1.9	2.3
25	RNA	434	7,322	23,764	55,950	51,147	23,779	11,479	3,797	987	51,691	12,175	57,595
	RIN	1.7	2.4	2.2	2.0	1.9	2.5	2.5	2.5	2.4	1.8	NM	2.1
26	RNA	7,197	22,423	26,426	36,685	46,714	73,221	22,950	3,085	672	52,705	343,366	118,509
	RIN	2.5	2.5	NM	2.2	NM	2.3	2.5	2.4	1.1	2.1	2.3	2.2
27	RNA	398	76	6,558	47,083	59,324	126,065	76,154	5,985	22,133	28,011	137	934
	RIN	2.6	5.1	1.2	2.0	1.9	2.4	2.4	2.4	2.4	2.5	3.1	4.9
28	RNA	25,430	19,610	185,703	152	56,276	259,012	11,032	38,413	24,973	66,103	1,307,318	150,052
	RIN	2.4	2.5	2.4	2.1	2.3	2.2	2.4	2.3	2.4	2.5	2.1	2.0

**Table 3:** Overall comparison of the four RNA extraction protocols for quantity (in pg/μl) and quality (RIN) for ten year old FFPE samples.

**Ten year old samples:**		**Protocol 1**	**Protocol 2**	**Protocol 3**	**Protocol 4**
**FFPE tissue input**		80 μm	10 μm	20 μm	50 μm
**Successful extractions**		36/42 (86%)	26/42 (62%)	38/42 (90%)	30/42 (71%)
**RNA (pg/μl)**	Minimum value	975	1,650	1,314	9864
	Maximum value	73,861	165,588	137,594	314,693
	Mean	15,964	51,985	29,661	107,042
	Median	8,750	31,104	19,458	86,682
**RIN**	Minimum value	1.0	1.5	1.0	1.8
	Maximum value	2.5	5.2	4.4	6.1
	Mean	1.8	2.5	2.3	2.4
	Median	2.0	2.3	2.2	2.2

**Table 4:** Overall comparison of the four RNA extraction protocols for quantity (in pg/μl) and quality (RIN) for recently archived months old FFPE samples.

**Months old samples:**		**Protocol 1**	**Protocol 2**	**Protocol 3**	**Protocol 4**
**FFPE tissue input**		80 μm	10 μm	20 μm	50 μm
**Successful extractions**		36/42 (86%)	34/42 (81%)	42/42 (100%)	35/42 (83 %)
**RNA (pg/μl)**	Minimum value	76	152	672	34
	Maximum value	185,703	259,012	115,194	2,209,739
	Mean	25,133	64,317	26,808	180,584
	Median	14,782	48,255	14,599	51,245
**RIN**	Minimum value	1.1	1.6	1.0	1.4
	Maximum value	5.1	2.7	2.6	4.9
	Mean	2.2	2.1	2.2	2.3
	Median	2.3	2.0	2.4	2.2

**Table 5:** Comparison of the four protocols for quantity and quality of extracted RNA for only samples that produced biologically-useful RNA (RIN ≥ 1.4) in ten year old FFPE samples. Case success is defined for each protocol as the percentage of samples where at least one of three RNA extraction attempts provided a RIN ≥ 1.4.

**Ten year old samples:**		**Protocol 1**	**Protocol 2**	**Protocol 3**	**Protocol 4**
**FFPE tissue input**		80 μm	10 μm	20 μm	50 μm
**Successful extractions**		26/42 (62%)	26/42 (62%)	35/42 (83%)	30/42 (71%)
**Case success**		12/14 (86%)	14/14 (100%)	13/14 (93%)	13/14 (93%)
**RNA (pg/μl)**	Minimum value	1,236	1,650	1,314	9,864
	Maximum value	73,861	165,588	137,594	314,693
	Mean	17,754	51,985	30,160	107,042
	Median	13,692	31,104	20,229	86,682
**Minimum RIN of 1.4**	Minimum value	1.4	1.5	1.9	1.8
	Maximum value	2.5	5.2	4.4	6.1
	Mean	2.0	2.5	2.3	2.4
	Median	2.1	2.3	2.3	2.2

**Table 6:** Comparison of the four protocols for quantity and quality of extracted RNA for only samples that produced biologically-useful RNA (RIN ≥ 1.4) in recently archived months old FFPE samples. Case success is defined for each protocol as the percentage of samples where at least one of three RNA extraction attempts provided a RIN ≥ 1.4.

**Months old samples:**		**Protocol 1**	**Protocol 2**	**Protocol 3**	**Protocol 4**
**FFPE tissue input**		80 μm	10 μm	20 μm	50 μm
**Successful extractions**		34/42 (81%)	34/42 (81%)	39/42 (93%)	35/42 (83%)
**Case success**		14/14 (100%)	14/14 (100%)	14/14 (100%)	14/14 (100%)
**RNA (pg/μl)**	Minimum value	76	152	987	34
	Maximum value	185,703	259,012	115,194	2,209,739
	Mean	24,818	64,317	26,843	180,584
	Median	14,781	48,255	15,146	51,245
**Minimum RIN of 1.4**	Minimum value	1.5	1.6	1.4	1.4
	Maximum value	5.1	2.7	2.6	4.9
	Mean	2.3	2.1	2.3	2.3
	Median	2.3	2.0	2.4	2.2

In the set of ten year old samples (Tables 1 and 5), protocols 1, 2, 3, and 4 extracted RNA with a minimum RIN of 1.4 in, respectively, 26, 26, 35, and 30 of the 42 triplicate RNA extraction procedures performed for each protocol. Thus, the success rate for extracting biologically useful RNA with protocols 1, 2, 3, and 4 was 62%, 62%, 83%, and 71%, respectively. When analyzed on a per sample basis, protocols 1–4 produced biologically useful RNA in at least one of three extractions for, respectively, 12, 14, 13, and 13 out of 14 cases in the set for a case success rate of, respectively, 86%, 100%, 93%, and 93%.

For the set of recently archived months old samples (Tables 2 and 6), protocols 1, 2, 3, and 4 extracted RNA with a minimum RIN of 1.4 in, respectively, 34, 34, 39, and 35 of the 42 RNA extraction procedures performed for each protocol, giving a success rate for extracting biologically useful RNA of 81%, 81%, 93%, and 83%, respectively. The case success rate was 100% for all four protocols; in other words, each protocol was able to extract RNA with a RIN ≥ 1.4 in at least one of the three attempts for every case in this set.

**Figure 1:**
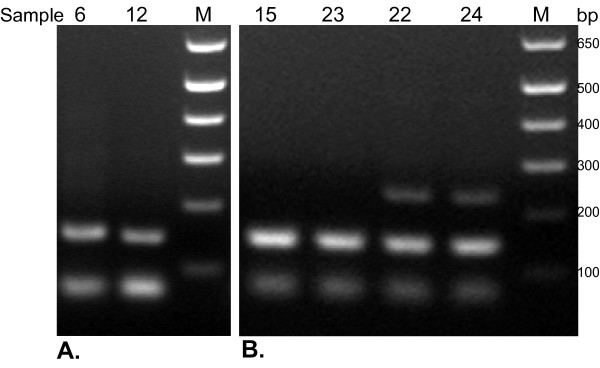
Amplification of G6PD by multiplex RT-PCR and analyzed by electrophoresis on 2% agarose gels. A. Representative examples of amplification products from RNA extracted from ten year old FFPE, samples 6 and 12; B. representative examples of amplification products from RNA extracted from recently archived months old FFPE, samples 15, 23, 22, 24. RNA fragments extracted from all 28 of the ten year old and months old samples were of sufficient size to generate shorter amplicons of G6PD 67 bp and 151 bp, visible on the gel; the 242 bp amplicon was visualized in none of the 10 year old samples and only six of 14 (43%) of the months old samples. All non-visualizing amplification products in the multiplex experiments were confirmed by PCR with single primer sets. M = marker (DNA ladder).

RT-PCR for the housekeeping gene glucose-6-phosphate dehydrogenase (6GPD) was performed using primers that targeted different fragment sizes of extracted RNA. All samples from both one and ten year old sets successfully amplified 67 bp and 151 bp fragments of G6PD mRNA. However, when primers targeting 242 bp fragments were tested, only six of 14 (43%) months old FFPE samples and none of the ten year old samples demonstrated G6PD gene expression (Figure 1).

## Discussion

Biopsies and surgical specimens are routinely fixed in formalin and embedded in paraffin for histological analysis. Although the morphological integrity of the tissues is preserved, this procedure causes degradation of nucleic acids, predominantly RNA. The development of protocols for RNA extraction from these samples makes it possible to molecularly study long-term archived tissues, opening new perspectives for research and diagnostic investigation. Soguero et al. were able to extract RNA in more than 20 year-old archival liver tissue [14]. In breast cancer, long-term follow-up is important because the time to developing distant metastases and death can vary greatly, with both recurrence time and death extending beyond 15 years after surgery [15-17]. Even patients with small tumors (stage T1N0M0) may have 10-year relapse-free survival rates of less than 75% in the absence of systemic therapy [18].

In addition to the impact of long-term storage, RNA can also be degraded during the relative delay associated with putting the specimen in fixative following surgical excision and by prolonged fixation prior to paraffin embedding [19]. However, the greater challenge is that RNA is chemically modified during formalin-fixation. Fortunately, some of the alterations can be reversed by heating the RNA to 50–55°C [2]. Many attempts have been made to avoid these chemical modifications by proposing use of alternative fixatives such as Bouin, Carnoy, acetone, and alcohol as substitutes for formalin [5,10,20]. These fixatives, however, introduce tissue artifacts that can make microscopic histopathological analysis difficult or even impossible. Moreover, these fixatives are not suitable for immunohistochemistry reactions, much more common in pathologic practice than gene expression analyses. Finally, samples in most archives are already fixed in formalin. So, for retrospective studies, it is imperative to develop and/or refine protocols for RNA extraction from FFPE.

The first step in RNA extraction from FFPE samples is deparaffinization of the sections obtained from the paraffin blocks. When deparaffinization is incomplete, the extracted RNA quality is worse [21]. In our work, in the set of 10-year-old samples, both the quantity and quality of RNA extracted with the deparaffinization solution d-limonene (protocols 2 and 3) were higher than that obtained using xylene – also known as xylol, dimethylbenzene, or methyltoluene – (protocol 1) (Tables 3 and 5). Due to other protocol differences, we cannot assess the impact of the deparaffinizing agent alone. It is worthy to note, however, that with the set of recently archived months old samples, the differences between protocols is smaller (Tables 4 and 6).

Historically, RNA integrity has been evaluated using agarose gel electrophoresis stained with ethidium bromide. Typically these gels show two peaks that correspond to ribosomal RNA species 28S and 18S. According to this technique, a given RNA sample is considered of good quality when the relation 28S:18S is equal to or higher than 2 [22]. The 2100 Bioanalyzer (Agilent Technologies, Santa Clara, CA) is a microfluidic platform that uses electrophoretic separation of RNA by molecular weight and provides laser-induced fluorescence measurements. Using electropherogram curves, 1208 RNA samples from different sources and in different degrees of degradation were analyzed and an algorithm was created to determine RNA integrity number (RIN). The RIN for a given sample ranges from 1 to 10, from totally degraded RNA to completely intact RNA [22]. The RIN method is superior to the 28S/18S ratio method for evaluating RNA quality in breast cancer tissue [23].

In frozen tissues, a RIN ≥ 6 provides more reproducible microarray results [23]. In FFPE samples, however, the RIN is much lower because RNA continues to degrade over time into small fragments. However, even low RIN RNA may still be used for some molecular analyses. Fragments of only 60 bp have been shown to successfully amplify in 80% of real-time RT-PCR reactions; even with FFPE samples stored long-term [24]. RNA extracted from 2- to 8-year-old FFPE produces RNA of sufficient quality for microarray analysis in at least 24% of unselected FFPE samples [25].

In frozen breast cancer tissue Strand et al. suggested a minimum RIN of 6 for gene expression analysis [23]. However, according to Madabusi et al., RNA with a RIN as low as 1.4 have been successfully used for gene expression analysis [12]. In our set of ten year old samples, using a minimum RIN of 1.4 as a threshold for RNA quality, only protocol 2 extracted RNA with a minimally acceptable quality for all 14 samples analyzed, 100% (Table 1). Protocol 1 extracted minimally acceptable RNA in 12 of 14 cases, 86% (failing to isolate minimally acceptable RNA for samples 6 and 7, even with three attempts). Protocol 3 extracted minimally acceptable RNA in 13 of 14 cases, 93% (failing for sample 7, even with three attempts). Protocol 4 extracted minimally acceptable RNA in 13 of 14 cases, 93% (failing for sample 4, even with three attempts) (Table 1). In the set of recently archived months old samples (Table 2), however, it was possible to extract RNA with a minimally acceptable quality in all 14 samples analyzed with all four protocols. These data indicate that ten year old RNA, and specifically RNA that is biologically assayable, could be isolated from every sample by at least one and usually most of the protocols. These data indicate that, if a chosen protocol fails to extract RNA from a given case in a first attempt, another attempt should be tried. However, in our series, if two initial attempts were unsuccessful, a third attempt using the same protocol was less likely to yield biologically usable results, especially for older samples. Thus, we recommend switching RNA isolation protocols after a second failed attempt. It would be unusual to completely exclude a case from molecular analysis because of insufficient or poor quality RNA.

Another important observation can be made in a careful analysis of Tables 1 and 2. There are discrepancies in both quantity and quality of extracted RNA among individual cases for each of the protocols. For example, in sample 7, with protocol 1, test 1, it was possible to extract 160,254 pg/μl of RNA. The quality of this RNA, however, showed complete degradation, without a measurable RIN. On the other hand, in sample 3, with protocol 1, test 1, only 1,236 pg/μl was extracted, but the RIN was 2.3. These data corroborate the observation made by Chung et al. that quantity and quality are independent parameters in RNA extraction [21].

For all parameters analyzed, both for quantity and quality, Protocol 1 did not perform as well as other protocols on our ten year old archived breast tissue, although it still produced sufficient RNA from most samples (Tables 1 and 3). Of the remaining three protocols (Table 3), protocol 2 extracted slightly higher quality RNA (RIN mean/median: 2.5/2.3) than protocols 3 and 4 (RIN mean/median: 2.3/2.2 and 2.4/2.2, respectively). However, protocol 4 extracted more RNA than the other protocols (mean of 107,042 pg/μl and median 86,682 pg/μl) (Tables 1 and 3). Protocol 4 used a total of 50 μm of tissue sections compared to 10 μm and 20 μm tissue sections used in protocols 2 and 3; it might be argued, therefore, that the reason protocol 4 extracted more RNA than protocols 2 and 3 was higher input of tumor tissue. However, protocol 1 had the highest input of tissue, 80 μm, yet extracted less RNA than the other protocols. Thus, the quantity of extracted RNA may or may not be related to tissue input. Protocol 2, which used only 10 μm of tissue input, may be a good choice when the amount of available tissue is scarce.

When we compared the results of the ten year old and recently archived months old samples in the extraction of biologically useful RNA (Tables 5 and 6), there were no significant differences in quality of extracted RNA between these different aged sample groups. The months old samples showed somewhat higher mean and median quantities of extracted RNA, except for Protocol 3, which extracted slightly higher amounts of RNA from the older tissues. All protocols, without exception, extracted biologically useful RNA in a higher percentage of attempts for months old compared to ten year old samples, although biologically useful RNA was still successfully extracted from each of the older samples a high percentage of the time.

Our RT-PCR data indicated that biologically useful RNA extracted from archivally stored FFPE tissue is suitable for RT-PCR analysis if the final product of amplification is designed to be 151 bp or less. We had a success rate of 100% for both the ten year old and more recently archived months old samples using primers for the housekeeping gene G6PD that amplified 67 bp and 151 bp. Successful amplification was achieved in none of our ten year old samples and in only 43% of our recently archived months old samples if the amplified fragment was 242 bp. This result is consistent with expected RNA degradation over time. However, the lower quantity of extracted RNA from ten year old samples that was used for the RT-PCR may also have contributed to the result. Liu *et al.*[26], who cleverly designed the primers also used in this study, reported successful RT-PCR of 91% and 76% for 67 bp and 151 bp fragments of G6PD mRNA, respectively, but only 5% for 242 bp fragments measured from archival histologic specimens of diverse tissues (including decalcified specimen from bone) between one and 15 years old. RT-PCR of genes other than G6PD or use of RNA extracted from FFPE from other tissue sources could potentially produce different results.

All four protocols were time-consuming (one working-day for protocols 1, 3, and 4; two days for protocol 2 because of an overnight Proteinase K digestion), but easy to use. We personally appreciated the speed and quantity of RNA extracted using Protocol 4.

## Conclusion

In conclusion, although the magnetic bead-based protocol extracted more RNA than spin column-based protocols, all protocols were able to extract RNA with a minimally acceptable quality from ten year old archived FFPE samples. Although the quality of RNA extracted from ten year old FFPE was similar to the RNA extracted from recently archived months old samples, the quantity, consistency and success rate was higher in the months old group. The RNA extracted from FFPE is suitable for RT-PCR analysis if the final product of amplification is up to 151 bp, with a success rate of 100% for both ten year old one and months old samples. If a chosen protocol fails to extract RNA from a given case in a first attempt, another extraction and then an alternative protocol should be attempted before excluding this case from further molecular analysis.

## Methods

### Samples

The study conformed to the ethical guidelines of the 1975 Declaration of Helsinki and was approved by the Ribeirão Preto local Ethics Committee, Brazil, and Stanford University. Twenty-eight paraffin blocks containing breast cancers diagnosed in 1997 (n = 14 samples, ten years old) and 2007 (n = 14 samples, three to ten months old) were retrieved from the archives of the Pathology Service of the General Hospital of Ribeirão Preto Medical School, University of São Paulo, Brazil. Each block contained one piece of breast cancer tissue measuring at least 1.5 × 1.5 cm in area. Using a conventional microtome (Leica RM2125), histological sections 10 microns in thickness were cut from each block according to protocol specifications (Table 7). A new sterile blade was used for each block to avoid contamination among the samples. All reactions were performed in an RNase-free environment; benches, instruments, and pipetters were cleaned and treated with RNaseZap solution (Ambion Inc., Austin, TX) before each reaction, and RNase-free tips and microtubes were used.

**Table 7:** Distinctions among the three column purification-based protocols used in this study.

Parameter	**Protocol 1**	**Protocol 2**	**Protocol 3**
**Histological sections**	4 sections of 20 μm (80 μm)	1 section of 10 μm (10 μm)	2 sections of 10 μm (20 μm)
**Deparaffinization**	1 ml xylene 100% (3 min – 55°C)	800 μl d-limonene (5 min – room temperature)	1 ml d-limonene (10 min – room temperature)
**Proteinase K incubation**	3 hours – 50°C	"*Overnight*" – 55°C	3 hours – 55°C
**RNA isolation**	Solution provide by kit 1	Solution provide by kit 2	0.875 μl mercaptoethanol in 125 μl buffer provide by kit 3
**DNase incubation**	30 min – 37°C	45 min – 37°C	15 min – 37°C
**RNA purification**	Solutions provided by the kit	Solutions provided by the kit	Solutions provided by the kit

### RNA extraction

All protocols were performed according to Manufacturer's instructions using their specified input of FFPE tissue sections (Table 7). Protocol 1 utilized the kit "RecoverAll™ Total Nucleic Acid Isolation Optimized for FFPE Samples" (Ambion Inc., Austin, TX) [27]. Protocol 2 utilized the "High Pure RNA Paraffin Kit" (Roche Applied Science, Indianapolis, IN) [28]. Protocol 3 utilized the "Absolutely RNA^® ^FFPE Kit" (Stratagene, La Jolla, CA) [29]. Briefly, these three protocols share six steps: histological sectioning of FFPE tissue blocks, deparaffinization, Proteinase K digestion, RNA isolation, DNase incubation and RNA purification. Table 7 highlights the main differences among the three column purification-based protocols used in this work.

Protocol 4 utilized the Agencourt^® ^"FormaPure™ Kit" (Agencourt, Beverly, MA) [30] followed by DNAse I digestion (Ambion Inc., Austin, TX). This kit utilizes Solid Phase Reversible Immobilization (SPRI^®^) paramagnetic bead-based technology to isolate RNA from a maximum input of 10 mg of FFPE tissue (according to manufacturer's instructions, we used five 10 μm sections from each paraffin block). Similar to other protocols, a reagent (lysis buffer provided by the kit) is added to melt the paraffin and de-crosslink nucleic acids, and Proteinase K is added to complete tissue digestion and inactivate nucleases (60 minutes incubation time at 55°C). Contrary to the other three protocols, binding buffer is added to facilitate immobilization of the nucleic acids to the surface of paramagnetic beads and separation is magnetically-performed. This protocol does not require centrifugation.

The RNA extraction from each of the 28 cases was done in triplicate: 84 extractions per protocol for each of the four protocols, for a total of 336 RNA extraction procedures.

### Evaluation of quantity and quality of the extracted RNA

The RNA extracted in triplicate from the 28 breast cancer samples using the four protocols was analyzed (n = 336) for quantity and quality. To rapidly quantify RNA, the Agilent 2100 Bioanalyzer (Agilent Technologies, Santa Clara, CA), a chip-based nucleic acid separation system, was used. The Bioanalyzer utilizes a combination of micro-fluidics, capillary electrophoresis, and fluorimetry to determine RNA length, distribution and concentration [22,23]. The RNA 6000 Pico Kit (Agilent Technologies, Santa Clara, CA) was used together with a standardized RNA ladder (Ambion, Austin, TX) for RNA analysis and quantification. The quantity of RNA was expressed in pg/μl (per the PicoChip) and the quality was expressed by the RNA integrity number (RIN). For both quantity and RIN, the following parameters were evaluated: minimum value, maximum value, mean value, and median value using the statistical software GraphPad Prism, version 4.0 (San Diego, CA). Only samples which had measurable RINs were included in the analyses. Case success was defined for each protocol as the percentage of samples in the 14 sample set in which at least one of three RNA extraction attempts provided biologically useful RNA with a RIN ≥ 1.4.

### RT-PCR for assaying RNA fragment size using different target lengths for glucose-6-phosphate dehydrogenase (G6PD)

To determine fragment sizes of RNA extracted from archival FFPE specimens, we targeted the housekeeping gene G6PD with different mRNA primer sets and performed RT-PCR as described by Liu *et al.*[26]. From each of the 28 FFPE samples included in this study (14 ten year old samples and 14 months old samples), extracted RNA with RINs greater than 1.4 were used for RT-PCR analyses.

All primers were designed by Liu et al. One sense primer (5'-GGC AAC AGA TAC AAG AAC GTG AA) and three antisense primers were used such that combinations of these primers would generate PCR amplicons of 67 bp (5'-CGC AGA AGA CGT CCA GGA T), 151 bp (5'-CCA GCT CAA TCT GGT GCA G), and 242 bp (5'-CCC TCA TAC TGG AAA CCC ACT), respectively. The sense primer was cleverly designed by Liu et al. to span two exons so that it would not anneal to or amplify genomic DNA.

Multiplex RT-PCR was carried out using Superscript™III One-Step RT-PCR with Platinum Tag system (Invitrogen, Carlsbad, CA) and optimized using Universal Human Reference RNA (Stratagene, La Jolla, CA) prior to testing samples. The template consisted of 0.2–2 μg of RNA from the months old samples, depending on RNA quantity extracted, or 100 pg of RNA from 10 year old samples. Each reaction contained 250 pmol of sense primer and 100 pmol of each antisense primer. The first reverse transcription step was performed at 50°C for 30 min. The following PCR steps was carried out using a ''hot-start and touch-down'' program of 94°C for 2 minutes, followed by 1 cycle of denaturing at 94°C for 30 seconds, annealing at 66°C (one degree down per cycle until 59°C is reached) for 30 seconds, and extension at 68°C for 45 seconds, and then 30 cycles of 94°C for 30 seconds, 58°C for 30 seconds, and 68°C for 45 seconds. A final extension was performed at 68°C for 10 minutes. PCR products were analyzed by electrophoresis on 2% agarose gels and visualized using ethidium bromide staining. Fragments that were not visible on the gel by multiplex RT-PCR were reassessed by PCR with single sets of primers using the first RT-PCR product as template.

## List of abbreviations

FFPE = formalin-fixed paraffin-embedded; G6PD = glucose-6-phosphate dehydrogenase; RIN = RNA integrity number; RT-PCR = reverse transcription-polymerase chain reaction

## Authors' contributions

ARS and SSJ conceived the study. ARS obtained the FFPE samples, performed the RNA extraction, and evaluated the quantity and quality of the extracted RNA. HZ optimized and performed the RT-PCR procedures. ARS and SSJ analyzed the data. ARS, HZ, and SSJ wrote the manuscript and approved the final manuscript.
